# Assessment of Physician Self-reported Knowledge and Use of Maryland’s Extreme Risk Protection Order Law

**DOI:** 10.1001/jamanetworkopen.2019.18037

**Published:** 2019-12-20

**Authors:** Shannon Frattaroli, Katherine Hoops, Nathan A. Irvin, Alexander McCourt, Paul S. Nestadt, Elise Omaki, Wendy C. Shields, Holly C. Wilcox

**Affiliations:** 1 Center for Gun Policy and Research, The Johns Hopkins Bloomberg School of Public Health, Baltimore, Maryland; 2 Department of Health Policy and Management, The Johns Hopkins Bloomberg School of Public Health, Baltimore, Maryland; 3 Department of Anesthesiology and Critical Care Medicine, The Johns Hopkins School of Medicine, Baltimore, Maryland; 4 Department of Emergency Medicine, The Johns Hopkins School of Medicine, Baltimore, Maryland; 5 Department of Psychiatry and Behavioral Science, The Johns Hopkins School of Medicine, Baltimore, Maryland; 6 Department of Mental Health, The Johns Hopkins Bloomberg School of Public Health, Baltimore, Maryland

## Abstract

**Importance:**

On October 1, 2018, Maryland’s extreme risk protection order (ERPO) law took effect. This was the first ERPO law in the United States to authorize clinicians to initiate a civil court process to temporarily prohibit people behaving dangerously and at risk of engaging in violence from purchasing and possessing firearms. This is the first publication reporting results from a survey of physicians about ERPOs.

**Objectives:**

To assess Maryland physicians’ knowledge, past use, and likely future use of ERPOs, and to identify barriers to physicians’ use of ERPOs and strategies to address those barriers.

**Design, Setting, and Participants:**

This survey study conducted at The Johns Hopkins Hospital in Baltimore, Maryland, surveyed physicians, including emergency medicine physicians, pediatricians, and psychiatrists, using a 15-question online instrument between June 15, 2019, and July 1, 2019. Data analysis was performed in July 2019.

**Exposure:**

Maryland’s ERPO law.

**Main Outcomes and Measures:**

Knowledge, use, and likely use of ERPOs, barriers to use, and strategies to address those barriers.

**Results:**

Ninety-two of 353 physicians invited (26.1%) completed the survey; 1 respondent reported having filed an ERPO petition. Sixty-six respondents (71.7%) described themselves as not at all familiar with ERPOs. After reading a brief description of the ERPO law, 85 respondents (92.4%) indicated that they encounter patients whom they would consider for an ERPO at least a few times per year. Fifty-five respondents (59.8%) reported that they would be very or somewhat likely to file an ERPO petition when they identify a qualifying patient. Respondents identified time as the main barrier to using ERPOs (not enough time to complete paperwork, 57 respondents [62.6%]; not enough time to attend hearing at courthouse, 64 respondents [70.3%]), followed by concern that filing an ERPO would negatively affect their relationship with the patient (36 respondents [39.6%]). Having a coordinator to manage the process (80 respondents [87.0%]), training (79 respondents [85.9%]), participating in court hearings remotely (68 respondents [73.9%]), and having access to legal counsel (59 respondents [64.1%]) were all selected by large majorities of respondents as strategies to address barriers to ERPO use.

**Conclusions and Relevance:**

Awareness of ERPOs among physicians in the sample was low. Physicians are treating patients who would qualify for an ERPO, and respondents in the sample indicated a willingness to use ERPOs. Training, providing access to legal counsel, designating a clinician to process petitions, and allowing clinicians to participate remotely in court hearings were strategies respondents identified to address barriers to ERPO use. These survey findings identify concrete solutions for addressing barriers to physician use of ERPOs.

## Key Points

**Question:**

What do Maryland physicians know about extreme risk protection orders (ERPOs), and what are the barriers and facilitators to ERPO use in clinical settings?

**Findings:**

In this survey study of 92 physicians, respondents’ knowledge of ERPOs was low, but after reading a brief description, most reported treating patients who would qualify for an ERPO and a willingness to use ERPOs. A clinical coordinator to process ERPO petitions, training, access to legal counsel, and the ability to participate in court hearings remotely would help address barriers to ERPO use.

**Meaning:**

Maryland physicians’ knowledge of ERPOs, barriers to ERPO use, and strategies for addressing identified barriers may provide important insights to better support ERPO use by physicians.

## Teaser

This survey study assesses Maryland physicians’ knowledge, past use, and likely future use of extreme risk protection orders, barriers to their use, and strategies to address those barriers.

## Introduction

As of November 2019, 17 states and the District of Columbia have enacted extreme risk protection order (ERPO) laws. Such laws allow specified groups (law enforcement in all states, and family in 14 states and the District of Columbia) to petition a court when an individual is behaving dangerously and is at risk of harming themselves or others, and to request that the individual be temporarily prohibited from purchasing and possessing firearms.^1,2^ By intervening when violence appears imminent—but before harm has occurred—ERPO laws create a tool to prevent firearm injury. Preliminary research suggests that ERPOs are being used to intervene in threats of mass violence^3^ and may be effective in preventing suicide.^4,5,6^

In October 2018, Maryland became the first US state to authorize physicians and other clinicians as petitioners under the ERPO law.^2,7^ Policy makers in the District of Columbia and Hawaii enacted ERPO laws in 2019 that also included clinicians, with effective dates in May 2019 and January 2020, respectively.^2,8,9^ Dangerous threats and behaviors that suggest suicidal intent are often revealed in the clinical setting.^10^ Patients may also threaten or disclose plans to engage in interpersonal violence.^11^ Authorizing clinicians to petition the court for an ERPO provides a concrete tool for removing access to guns when patients are at imminent risk of violence.

Uptake by clinicians in Maryland has been slow. Between October 1 and December 31, 2018, clinicians petitioned for 4 (1.3%) of the 303 ERPOs filed in Maryland courts.^12^ In Maryland, ERPO petitioners must complete a court form that includes details about the person and behaviors of concern and explain their request before a judge or commissioner, who determines whether to issue a temporary ERPO lasting a maximum of 7 days. The petitioner must return to court for a second hearing with the respondent for the judge to decide whether to extend the order for up to 1 year.^7^ We developed and fielded a survey study of emergency medicine, pediatrics, and psychiatry physicians in 1 medical institution to understand what they know about the ERPO law, whether they would use it, and what they need to support ERPO use.

## Methods

We designed a 15-question survey and invited physicians in emergency medicine, pediatrics, and psychiatry at The Johns Hopkins Hospital in Baltimore, Maryland, to participate via an email that included a survey link. We identified these departments as those most likely to have physicians who use ERPOs. Those who completed the survey opted whether to include their contact information for a drawing with several $25 gift cards as prizes. We invited participants with an email from the department chair or a recognized administrator and sent 1 reminder email. All physicians (attending and resident) from the 3 departments were eligible. We calculated response rates on the basis of the number of faculty and residents on each department’s roster. We emailed invitations on June 15, 2019, and closed the survey on July 1, 2019.

The Johns Hopkins Bloomberg School of Public Health institutional review board deemed this survey not human research; thus, informed consent was not needed. At the start of the survey, we provided a brief description noting that responses would be anonymous and reported in aggregate. This study follows the American Association for Public Opinion Research (AAPOR) reporting guideline.

### Statistical Analysis

 Data analysis was performed in July 2019. We used SAS statistical software version 9.4 (SAS Institute) to analyze the data. We did not test for statistical trends or significance.

## Results

Of the 353 physicians invited, 92 (26.1%) completed the survey. Response rates varied across the departments, with physicians from psychiatry most likely to respond (50 respondents [42.4%]), followed by emergency medicine (26 respondents [23.6%]), and pediatrics (16 respondents [12.8%]). One physician (a psychiatrist) in our sample reported having filed an ERPO petition; however, most respondents (66 [71.7%]) described themselves as not at all familiar with ERPOs. Psychiatrists reported higher levels of familiarity (17 respondents [34.0%]) relative to emergency medicine physicians (6 respondents [23.1%]) and pediatricians (3 respondents [18.8%]). After reading a brief description of ERPOs, almost all respondents (85 [92.4%]) indicated that they encountered patients for whom they would consider an ERPO petition at least a few times per year, with most emergency medicine physicians (18 respondents [69.2%]) reporting such encounters frequently. Respondents indicated they would be very or somewhat likely to file an ERPO petition when they identify a qualifying patient (55 [59.8%]), with greater willingness from emergency medicine physicians (17 [65.4%]) and psychiatrists (31 [62.0%]) compared with pediatricians (7 [43.8%]) (Table 1).

**Table 1.** Respondents’ Familiarity With Maryland’s ERPO Law and Their Opportunity and Likelihood of Use, by Specialty

Question	Respondents, No. (%)			
	Emergency Medicine (n = 26)	Pediatrics (n = 16)	Psychiatry (n = 50)	Total (N = 92)
How familiar are you with ERPOs?				
Very familiar	2 (7.7)	0	2 (4.0)	4 (4.3)
Somewhat familiar	1 (3.8)	0	5 (10.0)	6 (6.5)
A little familiar	3 (11.5)	3 (18.8)	10 (20.0)	16 (17.4)
Not at all familiar	20 (76.9)	13 (81.3)	33 (66.0)	66 (71.7)
How often do you estimate you encounter a patient at extreme risk of violence or suicide who you would consider for an ERPO?				
Daily	3 (11.5)	0	0	3 (3.3)
Weekly	9 (34.6)	0	2 (4.0)	11 (12.0)
Monthly	6 (23.1)	2 (12.5)	10 (20.0)	18 (19.6)
A few times per year	8 (30.8)	11 (68.8)	34 (68.0)	53 (57.6)
Never	0	3 (18.8)	4 (8.0)	7 (7.6)
How likely would you be to file a petition against a patient at extreme risk of violence or suicide?				
Very likely	4 (15.4)	1 (6.3)	10 (20.0)	15 (16.3)
Somewhat likely	13 (50.0)	6 (37.5)	21 (42.0)	40 (43.5)
Somewhat unlikely	5 (19.2)	6 (37.5)	14 (28.0)	25 (27.2)
Very unlikely	4 (15.4)	3 (18.8)	5 (10.0)	12 (13.0)

Abbreviation: ERPO, extreme risk protection order.

Time was the main barrier to filing an ERPO (not enough time to complete paperwork, 57 respondents [62.6%]; not enough time to attend hearing at courthouse, 64 respondents [70.3%]), with emergency medicine physicians most likely to indicate that the time to complete the paperwork (20 of 26 respondents [76.9%]) or attend hearings (23 of 26 respondents [88.5%]) would keep them from using ERPOs. Pediatricians and psychiatrists also cited time as important (13 of 16 [81.3%] and 32 of 50 [64.0%], respectively). Concern that filing an ERPO would negatively affect their relationship with the patient was noted (36 respondents [39.6%]), with psychiatrists (26 respondents [53.1%]) and pediatricians (7 respondents [43.8%]) more likely than emergency medicine physicians (3 respondents [11.5%]) to identify this as a barrier. Having a coordinator to manage the process (80 respondents [87.0%]), training (79 respondents [85.9%]), participating in court hearings remotely (68 respondents [73.9%]), and having access to legal counsel (59 respondents [64.1%]) were all strategies to address these barriers selected by large majorities of respondents. Pediatricians were 4 times more likely than emergency medicine physicians or psychiatrists to express that clinicians should not file ERPO petitions (3 respondents [18.8%] vs 1 respondent [3.8%] and 2 respondents [4.1%], respectively) (Table 2).

**Table 2.** Barriers and Facilitators to Physicians’ ERPO Use

Question	Respondents, No. (%)			
	Emergency Medicine (n = 26)	Pediatrics (n = 16)	Psychiatry (n = 50)	Total (N = 92)
What barrier(s) prevent you from being able to file an ERPO petition? Check all that apply.^a^				
Not enough time to complete paperwork	20 (76.9)	11 (68.8)	26 (53.1)	57 (62.6)
Not enough time to attend hearing at courthouse	23 (88.5)	11 (68.8)	30 (61.2)	64 (70.3)
Not a billable service	3 (11.5)	1 (6.3)	6 (12.2)	9 (9.9)
It may negatively affect my relationship with the patient	3 (11.5)	7 (43.8)	26 (53.1)	36 (39.6)
I don’t think clinical providers should file ERPO petitions	1 (3.8)	3 (18.8)	2 (4.1)	6 (6.6)
Other	9 (34.6)	6 (37.5)	17 (34.7)	32 (35.2)
What tool(s) would help you file an ERPO petition? Check all that apply.				
Training on ERPO	22 (84.6)	16 (100.0)	41 (82.0)	79 (85.9)
Consultation with legal expert	19 (73.1)	10 (62.5)	30 (60.0)	59 (64.1)
A trained coordinator to complete and follow through the petition	25 (96.2)	15 (93.8)	40 (80.0)	80 (87.0)
Remote court hearings (ie, can join by phone)	21 (80.8)	8 (50.0)	39 (78.0)	68 (73.9)
Other	3 (11.5)	1 (6.3)	2 (4.0)	6 (6.5)

Abbreviation: ERPO, extreme risk protection order.

^a^ Ninety-one participants responded to this item.

## Discussion

The low number of clinicians filing ERPO petitions in Maryland is concerning given that physicians in our sample reported treating patients who meet ERPO criteria and that most would likely use the law when encountering such a patient. These survey findings point to several concrete strategies to support physician use of ERPOs. Training and access to a legal expert are essential. The need for legal expertise may be particularly salient when the patient at risk is a minor.

Addressing physicians’ availability to petition for these orders and to attend hearings is also necessary. Designating a coordinator to process petitions resonated with survey respondents. In Maryland, authorized ERPO petitioners include certain counselors, nurses, and therapists, as well as all physicians, psychologists, and social workers, any of whom could serve in a coordinating role. How clinicians would work with an ERPO coordinating clinician while complying with patient privacy requirements and meeting court standards when presenting cases are important issues. Most respondents also supported allowing clinicians to participate remotely in court hearings. The legal and logistical requirements for remote participation would need to be addressed to ensure due process.

As states move forward with ERPO implementation, the early experience of Maryland demonstrates the importance of attention to clinicians’ needs. Ensuring that implementation accommodates clinicians’ realities is critical. These survey findings provide initial insight into the early experience implementing ERPOs within 1 medical institution and offer guidance for how to maximize ERPO policies that include clinician petitioners.

### Strengths and Limitations

A strength of this study is that, to our knowledge, it is the first published survey data exploring what physicians know about ERPOs, how they are using or might use ERPOs, barriers to their use in clinical settings, and what they need to implement them. The study also has limitations. These findings are from a sample of physicians from 1 institution. The response rate was low (26.1%), particularly for pediatricians (12.8%). The survey was not designed to be generalizable. Our small sample precluded statistical testing for differences among clinicians in terms of training and years of experience. We did not explore how differences in inpatient and outpatient clinical settings may be associated with physicians’ willingness to petition for an ERPO. Because of differences in the therapeutic relationship, there is a need to understand whether physicians view ERPOs differently according to specialty and treatment setting.

## Conclusions

Extreme risk protection orders may be promising tools for reducing the risk of violence among people who are behaving dangerously and at imminent risk of engaging in violence. Attention to implementation within clinical settings is needed. This survey identifies concrete solutions for addressing implementation barriers for clinicians filing ERPO petitions.
